# Clinical Features of Takotsubo Syndrome and Its Differential Diagnostic Criteria in Clinical Nursing Practice: A Review of the Literature

**DOI:** 10.1007/s11886-023-01905-7

**Published:** 2023-07-19

**Authors:** Pierluigi Lezzi, Roberto Lupo, Noemi Cimarelli, Luana Conte, Giorgio De Nunzio, Stefano Botti, Alessandro Calcagnile, Ciro Del Coco, Antonino Calabro, Ivan Rubbi, Maicol Carvello, Elsa Vitale

**Affiliations:** 1 ‘Veris Delli Ponti’ Hospital, ASL (Local Health Authority), Scorrano, Italy; 2 San Giuseppe da Copertino Hospital’, Local Health Authority Lecce, Lecce, Italy; 3 Freelance nurse, Lecce, Italy; 4 Laboratory of Biomedical Physics and Environment, Department of Mathematics and Physics “E. De Giorgi”, University of Salento, Lecce, Lecce, Italy; 5 Laboratory of Interdisciplinary Research Applied to Medicine (DReAM), University of Salento and ASL (Local Health Authority) Lecce, Lecce, Italy; 6 Hematology Unit, Azienda USL-IRCCS, Reggio Emilia, Italy; 7 Nuovo Ospedale Degli Infermi’ Hospital, ASL (Local Health Authority), Biella, Italy; 8 School of Nursing, University of Bologna, Bologna, Italy; 9 Community Hospital’, ASL (Local Health Authority) of Romagna, Ravenna, Italy; 10 Department of Mental Health, ASL (Local Health Authority), Bari, Italy

**Keywords:** Nurse, Takotsubo, Emergency room, Emergency, Nursing role

## Abstract

**Purpose of Review:**

Takotsubo cardiomyopathy (TCM) is a heart disease that mimics the symptoms of a myocardial infarction (MI). The exact cause of TCM is unknown, but the main theory is that the syndrome is triggered by an excessive release of catecholamines, a consequence of factors related to stress or severe emotional distress. The aim of this review is to summarize the various scientific journal articles on the nursing differential diagnosis of TCM, on the specific nurse training (particularly the role of the Advanced Practice Nurse, APN), and on the nursing educational support for the patient after hospital discharge.

**Recent Findings:**

A literature review was conducted on Medline (via PubMed), Web of Science (WoS), Scopus, and Google Scholar databases. Relevant indexed articles that investigated the elements characterizing TCM in nursing differential diagnosis and the role of the APN were identified.

**Results:**

Sixteen studies were included in the review; they highlighted the role of the nurse in identifying and educating patients with TCM.

**Summary:**

Nurses must have a thorough understanding of the syndrome, the onset symptoms, the unusual characteristics, and the probable etiology of TCM in order to recognize and promptly treat patients affected by this syndrome and have the opportunity to educate them after hospital discharge to reduce the possibility of recurrence.

**Graphical Abstract:**

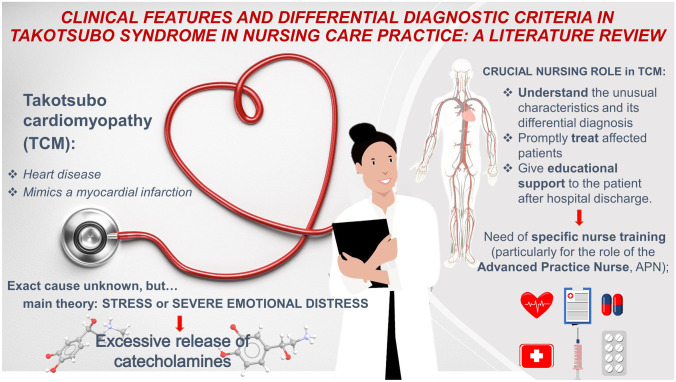

## Introduction

Takotsubo cardiomyopathy (TCM), also called stress cardiomyopathy (SC) or stress-induced cardiomyopathy, is a reversible heart disease that mimics the symptoms of an acute myocardial infarction (MI or AMI). An MI is the result of ischemia due to blockage of the coronary vessels, whereas in TCM there is no blockage of the vessels [1–3]. It is an atypical pattern of abnormalities of parietal kinetics such as excessive anomalous movement of the basal wall with medio-ventricular and apical hypokinesia [4]. These findings occur in the absence of obstructive coronary disease, and optimal management has not yet been established for either physicians or nurses [5]. The hallmark of the syndrome is a transient contractile abnormality of the left ventricle that causes a balloon-shaped morphology, which can be detected with left ventricular angiography or contrast-enhanced echocardiography. The left ventricle damage causes a contractile defect of the heart apex. During systole, or ventricular contraction, ventricular imaging shows a rounded hypokinetic apex with a narrow and hyper-contracted base [6]. The disease name is due to the resemblance of the characteristic left ventricular (LV) apical ballooning to the traditional Japanese octopus-fishing trap called takotsubo. The image on the echocardiogram of the heart appears with a wide base and a long, thin neck [1, 3].

The exact cause of Takotsubo cardiomyopathy is not known, but the main theory is that the syndrome is triggered by excessive release of catecholamines. The human body releases catecholamines during an acute stress response. Common triggers of this disease include hearing tragic news, such as a death or loss, receiving a diagnosis like cancer or other adverse pathologies, undergoing major surgeries, and in some cases, exercise or excitement [1]. It has been found that patients with SC have elevated levels of circulating catecholamines, from 7 to 34 times the normal value, compared to 2 or 3 times the normal value for patients with an acute MI. The chest pain in takotsubo cardiomyopathy mimics MI. This symptom has been associated with the ischemic effects of coronary vasospasm induced by catecholamines. Although stenosis or other lesions are generally not found on coronary angiography, in 2% of patients with takotsubo cardiomyopathy, altered blood flow due to coronary vasospasm has been observed [7]. This response results in decreased cardiac contractility and significantly reduces the heart ability to pump and circulate blood volume. The left ventricular tract responds inappropriately to catecholamine overload by narrowing, with an obstruction to blood flow. When this change is associated with a balloon-shaped apex of the left ventricle, it becomes difficult for the ventricle to completely empty. As a result, blood accumulates and increases the volume of the heart against the ventricular wall. The consequence is reduced contractile state, with decreased cardiac output, and increased myocardium oxygen demand [3].

TCM has been known since the early 1990s and usually affects postmenopausal women [8, 9]. The age range of patients with TCM is between 58 and 75 years old, and about 90% of cases occur in women. The large discrepancy in prevalence between men and women has long been established, but the explanations for this are contradictory, and no clear pathophysiological explanation has been established [10]. Other causes of the syndrome include ethnic or racial variations that occur predominantly in the Japanese population, hormonal influences due to the high percentage of middle-aged female victims, and a potential genetic predisposition in siblings or patients with a type 1 CD36 disorder (platelet glycoprotein 4) [11]. Cases of insect bites, especially bee stings, have been identified as causing a combination of anaphylactic mediators along with endogenous catecholamines, triggering severe stress resulting in TCM [12]. Excessive circulating catecholamines have been implicated in the pathophysiology of this condition, which is very similar to what can occur in patients with adrenal and extra-adrenal pheochromocytoma, making it an additional identified cause of the syndrome [13].

The connection between stress and illness has been part of popular wisdom for a long time, and even our common language has phrases that describe this condition, such as “scared to death” and “broken heart” [1]. Undoubtedly, TCM represents a serious and potentially lethal disorder because it can lead to serious complications. Research shows that 10% of TCM patients develop ventricular wall rupture, cardiogenic shock, and malignant arrhythmia, with a mortality rate of approximately 8% [4].

Nurses should be prepared to provide education and counseling to patients who might receive this diagnosis, which is possible especially when (a) the individuals present signs and symptoms of MI, (b) they appear to be at low risk for coronary artery disease, but (c) have experienced a stressful event prior to the onset of signs and symptoms [6]. Initially, TCM is often indistinguishable from MI, which is why nursing management and recognition of signs and symptoms with differential diagnosis are essential to improve final health outcomes. The nurse with specific advanced skills and adequate training can definitely, as part of a multidisciplinary team, impact the final treatment outcomes.

This article aims to present and review the physiology of TCM, its clinical characteristics, and diagnostic criteria, with particular attention to nursing differential diagnosis, specifically focusing on the signs and symptoms that nurses should observe in TCM patients.

## Materials and Methods

**Fig. 1:**
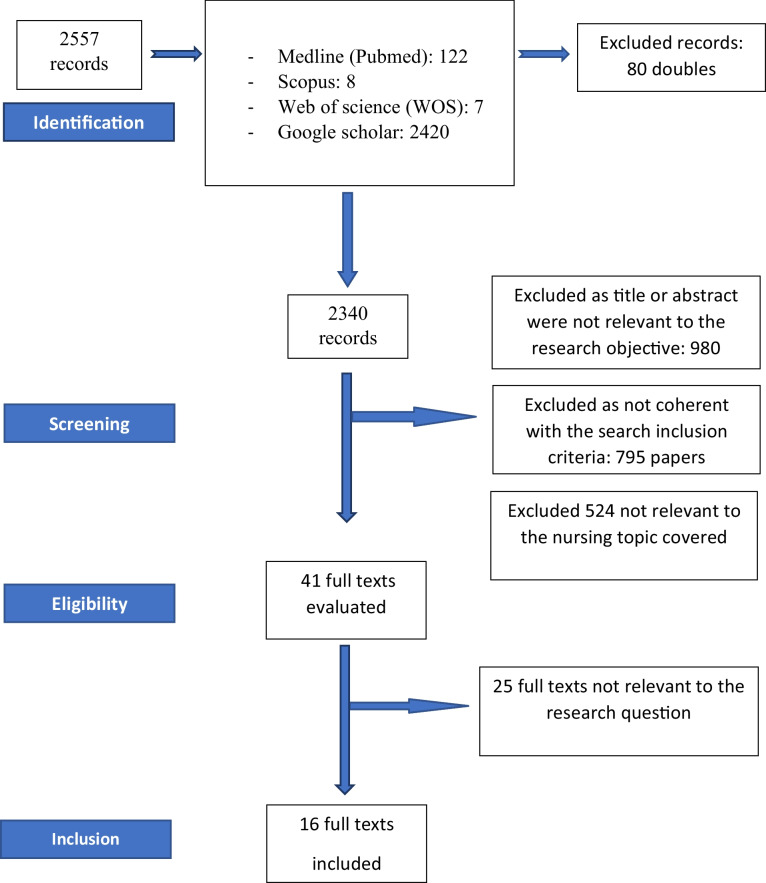
Prisma (2009) Search flowchart

To conduct the review, a research question was formulated using Population, Intervention, and Outcome (PIO) as the methodology (Table 1). Bibliographic consultation was carried out using the MEDLINE (via PubMed), Scopus, Web of Science (WOS), and Google Scholar databases. Articles written both in English and Italian were considered. The search strategy involved the use of the following terms, both for free search and for using individual MeSH terms and in combination with boolean operators AND and OR: “takotsubo,” “nurse,” “nursing role,” “emergency room,” “emergency” (Table 2). Through an initial electronic bibliographic search of databases, all articles considered relevant were identified for the purposes of this review. Duplicate articles were removed, and suitable articles were subsequently identified. In particular, two authors evaluated studies that were potentially relevant to the initial research objective and with the possibility of accessing full-text articles (Fig. 1). This review included studies that met the following criteria:

- diagnosis of TCM syndrome;
- full-text versions available in English or Italian;
- role of the Advanced Practice Nurse in TCM syndrome;
- role of the nurse in TCM syndrome;
- distinguishing characteristics of nursing differential diagnosis in TCM and IMA.

**Table 1:** The PIO tool for conducting literature review

Population	Patients with TCM
Intervention	Nursing interventions in TCM syndrome
Outcomes	Differential nursing diagnosis for IMA and TCM with improved disease management and reduction of medical and nursing treatment errors

*TCM* Takotsubo cardiomyopathy; *IMA* myocardial infarction, *PIO* population, intervention, outcomes, *APN* advanced practice nurse

**Table 2:** Combination of keywords used with boolean operators

***Database***	***String***	***Limits***	***Results***
Medline (PubMed)	*Search: ****(takotsubo) AND (nursing role)*** *(“takotsubo”[All Fields] OR “takotsubo s”[All Fields]) AND ((“nursing”[MeSH Terms] OR “nursing”[All Fields] OR “nursings”[All Fields] OR “nursing”[MeSH Subheading] OR “nursing s”[All Fields]) AND (“role”[MeSH Terms] OR “role”[All Fields]))*	None	*7*
Medline (PubMed)	Search: **(takotsubo) AND (nurse)** (“takotsubo”[All Fields] OR “takotsubo s”[All Fields]) AND (“nurse s”[All Fields] OR “nurses”[MeSH Terms] OR “nurses”[All Fields] OR “nurse”[All Fields] OR “nurses s”[All Fields])	None	18
Medline (PubMed)	Search: **(emergency room) AND (tako tsubo)** (“emergency service, hospital”[MeSH Terms] OR (“emergency”[All Fields] AND “service”[All Fields] AND “hospital”[All Fields]) OR “hospital emergency service”[All Fields] OR (“emergency”[All Fields] AND “room”[All Fields]) OR “emergency room”[All Fields]) AND (“tako”[All Fields] AND “tsubo”[All Fields])	None	15
Medline (PubMed)	Search: **(tako tsubo) AND (emergency)** “tako”[All Fields] AND “tsubo”[All Fields] AND (“emerge”[All Fields] OR “emerged”[All Fields] OR “emergence”[All Fields] OR “emergences”[All Fields] OR “emergencies”[MeSH Terms] OR “emergencies”[All Fields] OR “emergency”[All Fields] OR “emergent”[All Fields] OR “emergently”[All Fields] OR “emergents”[All Fields] OR “emerges”[All Fields] OR “emerging”[All Fields])	None	82
Scopus	(Title-abs-key (takotsubo AND nurse) AND title-abs-key (emergency room)	None	8
Google Scholar	Takotsubo and nurse and emergency room AND nursing role	Scientific articles	2420
Web of Science (WOS)	Takotsubo (All Fields) and nurse (All Fields)	None	7

Studies excluded from the review met the following criteria:

- studies related to myocardial infarction (IMA);
- studies related to medical interventions and prescriptions rather than nursing management in TCM syndrome;
- patients who did not receive any nursing education and training on TCM syndrome;
- studies that did not investigate the elements that distinguish the nursing differential diagnosis;
- pediatric population.

## Results

Table 3 reports the 16 articles comprised in the review, including authors, year of publication, quantity and type of patients, evaluation of the parameters studied, objectives, and results. A prospective study of 65 menopausal patients demonstrated that survival with TCM is superior to that of acute myocardial infarction (AMI).

**Table 3:** Main features of the selected studies

**Author, year**	**Objective**	**Design**	**Sample, setting**	**Main result**
***PubMed database***				
Vriz et al. [2]	To describe the actual incidence and prevalence among patients with acute coronary syndrome, and the recurrence rate of TCM in the emergency department of a community hospital	Observational, prospectic	25 TCM patients	This study highlights an increase in the incidence rate of TCM in the general population, with a higher prevalence of TCM recurrence rate compared to data reported in previous studies
Cantey [16]	To describe the story of a woman who is diagnosed with TCM	Observational, case report	Woman, 71 y.o	Description of TCM syndrome and the evolution of the pathology in the woman considered in the case report
Ripa et al. [9]	Differential diagnosis recognition of TCM vs an acute myocardial infarction (MI)	Observational, case report	Woman, 78 y.o	Based on the limited increase in cardiac enzymes, the absence of coronary lesions, and the presence of typical echocardiographic changes, the Authors suspected a TCM syndrome
Strachinaru et al. [15]	Diagnosis of TCM in a woman with only an increase in cardiac troponin	Observational, case report	Woman, 83 y.o	TCM recognition in a woman with only increased cardiac troponin without symptoms corresponding to the syndrome
Padilla et al. [7]	Examination of the pathophysiology, signs and symptoms, diagnosis, and treatment of TCM	Observational, descriptive	22 subjects with TCM	List of signs and symptoms of TCM and description of some favorable patient outcomes
Massobrio et al. [14•]	To evaluate the mortality rate and all-cause survival in a series of female patients with TCM compared to STEMI (ST-Segment Elevation Myocardial Infarction) patients of the same age and sex during follow-up	Observational, prospectic	65 subjects with TCM	The TCM group had a lower mortality rate than the IMA group, which seems to suggest that TCM and IMA are two different clinical entities with different clinical outcomes
***Scopus database***				
Sharkey et al. [13]	Comprehensive diagnostic investigations for the unexpected discovery of a catecholamine-producing pheochromocytoma	Observational, descriptive	2 male subjects of different age: 16 and 66 y.o	Observations show a common link between the occurrence of TCM and elevated catecholamine levels in both male and female patients with the presence of pheochromocytoma
***Web of Science WOS database***				
Nyeche and Winokur [4]	Clinical manifestations of TCM	Observational, descriptive	Samples from different studies compared	Elements characterizing the diagnosis of TCM, especially cases of previous surgery
Griffin and Logue [3]	To describe the diagnostic criteria used for TCM	Observational, descriptive	22 women with TCM	Factors involved in the diagnosis of TCM in middle-aged women
Dahlviken et al. [5]	To describe the experiences of women with TCM cardiomyopathy from a short-term perspective	Observational, qualitative	14 women with TCM	Women suffering from TCM may be a target group for holistic and individual health care with a longer duration of follow-up
***Google Scholar database***				
Wallström et al. [10]	Describe and interpret patients' narratives of the long-term stress experienced before the onset of TCM syndrome	Observational, qualitative	19 subjects with TCM	Long-term stressful circumstances can cause vulnerability to acute psychological or physical stressors and, subsequently, the onset of TCM syndrome
Sundelin et al. [8]	Describe the stress before and after the onset of TCM syndrome	Observational, mixed method	20 subjects with TCM	Patients with TCM syndrome reported long-term stress at times with an acute stress trigger before the onset of TCM syndrome. Stress decreased over time but remained high for a considerable number of patients
Brenner and Powers [11]	Describe TCM syndrome on social stressful events	Observational, case report	Woman, 77 y.o	Widowed woman with stressful event: emergency room course is described
Swenson et al. [1]	To review the physiology, diagnostics, treatment, and complications of TCM and to report a case study of a patient who developed TCM	Observational, review	Supporting information was obtained through the survey of academic articles on TCM over the past decade	APN is crucial in differentiating between TCM and acute myocardial infarction, heart failure, pulmonary edema, dynamic outflow obstruction, and cardiogenic shock
Derrick [6]	To describe the clinical manifestations of TCM	Observational, descriptive	Man, 65 y.o	Timely recognition of TCM results in fewer errors and consequences for the patient
Ghanim et al. [12]	To describe TCM as a result of insect bite	Observational, case report	Woman, 37 y.o	Therapeutic treatment after insect bite of a woman with TCM

*PRISMA* Preferred Reporting Items for Systematic reviews and Meta-Analyses, *APN* advanced practice nurse

A study conducted through interviews with 19 people with TCM found that stressful life circumstances were characterized by experiences of excessive demands on daily life and eternal responsibilities, being treated unfairly, not getting what they were entitled to, and not being able to control or escape from the situation they were living in [10].

Further studies report cases of patients presenting to the emergency department whose diagnosis was confused with AMI due to similar symptoms, relating to chest pain and dyspnea; it has been shown that diagnostic and treatment errors are often present in these situations, and that healthcare personnel training is necessary to prevent emergency situations with non-specific and inadequate treatments [1, 6, 11, 15].

The advanced practice nurse (APN) is crucial in differentiating TCM from acute myocardial infarction (AMI), heart failure, pulmonary edema, dynamic outflow obstruction, and cardiogenic shock. Prompt recognition and intervention for patients with TCM are priorities for nurses, especially in emergency contexts such as the emergency department and other settings such as the coronary intensive care unit; moreover, the APN communicates to other present nurses the eventuality of TCM if, through their specific training, they recognize it [1, 5, 9].

Nursing education, after discharge, plays a fundamental role for TCM as patients must receive the right indications from healthcare providers to avoid recurring forms, thanks also to the help of family members [3, 11].

Qualitative interviews were conducted in which patients were encouraged to freely talk about their experience of stress, before, during, and after the onset of TCM. Such studies addressed the theme of stress from a subjective point of view and related to the intimate sphere of the subjects, revealing that stressful events are the direct cause of TCM and of the increase of catecholamines in the bloodstream [8, 12].

Maintaining a healthy lifestyle, seeking help in case of stressful events, and being aware of signs and symptoms of a cardiac event may be the best recommendations. One thing to keep in mind regarding TCM is that there are no specific reasons or evidence as to why a stressful event triggers signs and symptoms while others do not [7, 16].

## Discussion

TCM is a nosological entity that is much less rare than previously thought, as it is now diagnosed in about 2% of patients hospitalized for myocardial infarction. Prolonged perceived psychosocial stress is a component of the onset of TCM, potentiated by the gender identity attributed by society’s structure that wears down the defenses of individuals and creates vulnerability to an acute stressor [10]. This syndrome is a pathology that is attracting increasing interest due to its insidious and unrecognizable nature. It often makes diagnosis difficult due to the similarity of its symptoms with the clinical presentation of an MI [2, 9]. The aim of the review was to identify nursing diagnosis in relation to TCM, especially the identification by nurses of signs and symptoms to avoid confusing TCM with an MI.

As TCM is stress-induced and a recently identified syndrome [11], there are no double-blind randomized studies to identify the best nursing practices for identification. Nurses should have in-depth knowledge of the symptoms of TCM to anticipate therapeutic medical treatment, recognizing signs, and triggering factors [1]. The long-term prognosis is favorable. Patients should follow their healthcare providers’ recommendations regarding the physical and psychological aspects of the syndrome. With a full understanding of takotsubo cardiomyopathy, nurses can help patients heal their broken hearts [3, 7].

Specialist training of the advanced practice nurse (APN) is of fundamental importance, especially in a pathology where patient symptoms can be confused with others [11]. Several studies [2, 9, 15] show specific cases of TCM in patients arriving in the emergency room where the first approach and diagnosis are offered by the nursing staff, who play an important role in differentiating diagnoses and recognizing the syndrome compared to MI. Specialist training of APNs considers a fundamental theme for TCM, namely, the signs and symptoms that distinguish TCM from an MI. An accurate interview by the nurse of the elements characterizing TCM (including symptoms or events from the preceding days) makes recognition of TCM optimal (Table 4).

**Table 4:** Differentiation for nursing diagnosis between TCM and IMA

***Defining characteristics for nursing diagnosis in the differentiation between TCM and IMA***	***Takotsubo cardiomyopathy (TCM)***	***Acute myocardial infarction (IMA)***
*Symptoms*	• Sudden onset of chest pain [6] • Initial symptom is often dyspnea [6]	Thoracic pain, chest pressure, pain in the arms, shoulder, neck, or jaw [4]
*Onset*	• Emotional stress or period of increased physical activity	Blood clot/plaque causing cardiac ischemia [7]
*Nursing history/anamnesis*	• History of TCM [13] • Menopause [3] • History of hypertension, hyperlipidemia, and/or atrial arrhythmias [13] • History of previous stroke, gastric bleeding, chronic obstructive bronchopneumopathy [16] • Insect bites [12] • History of pheochromocytoma (rare tumor of the adrenal glands) [13] • History of platelet glycoprotein disorder [11] • Bad financial news [7] • Announcement of tragic news (e.g., cancer diagnosis, death of a family member, natural disasters) [4] • Collisions with motor vehicles [7] • History of previous surgeries [4] • Administration of catecholamine therapy [16] • Use or abstinence from illegal drugs [7] • Absence of atherosclerotic coronary disease or other pathological conditions to explain the observed pattern of temporary ventricular dysfunction (e.g., viral myocarditis) [14•]	• Family history of CAD, heart disease, diabetes, hypertension, smoking, unhealthy LDL levels, dietary fat. Age > 65 years [8]
*ECG reading*	• Transient elevation of the ST segment or inversion of the T wave prolonged QT interval [1] • Malignant ventricular arrhythmias [1]	ST-segment elevation or depression. Reversal of T wave. Prolonged QT interval associated with cardiac death [1]
*Blood biomarkers to be reported to the medical specialist*	• Small, rapid increase in CPK and/or blood levels of troponin 1 [3]	Troponin 1 and troponin increased. Increased levels of CPK and CK-MB [5]

*CAD* coronary artery disease, *CPK* creatine phosphokinase, *CK-MB* creatine kinase MB (blood) isoenzyme, *ECG* electrocardiogram, *LDL* low-density lipoproteins

In some hospital settings, the figure of the APN is also present in coronary intensive care units and emergency departments, where complete knowledge of ECG interpretation is essential for continuous cardiac monitoring of patients, as changes in the cardiac trace, specifically atrioventricular third-degree blocks and arrhythmias, are the first signs of worsening of the condition [12]. APNs must promptly recognize patients who have risk factors for TCM and communicate any anomalies to the other nurses present in coronary intensive care or emergency departments so that they can be prepared for the management of the pathology [1, 11].

The nature of the nursing relationship in patient interviews is holistic, seeing the person as a whole; it is based on the perspective of recognizing the individual subjective experience regarding health beliefs and values. This concept addresses interconnected elements of body, mind, emotion, spirit, socio-culture, relationships, context, and environment that are closely related to specific nursing training for TCM [5]. Therefore, the nurse, during the history-taking moment, should encourage verbalization of feelings to obtain an understanding of the patient’s perceptions, interview family members, friends, or close people who could contribute important information to recognize signs and symptoms of TCM, thus preventing nursing differential-diagnosis errors [1, 6]. The nursing assessment of data collection is of fundamental importance for medical diagnosis; timely recognition of signs and symptoms allows for avoiding errors that would result in inadequate treatment for the patient [1, 4]. The expectation of a complete recovery will reassure patients and their families, and therefore they should be instructed on the importance of outpatient follow-up to repeat the echocardiogram and confirm the resolution of TCM. This will be the place where the nurse and the doctor can collect the patient’s emotions, feelings, and anxieties again to prevent possible relapses [6].

## Limitations

The limitation of this review concerns the scarcity of scientific literature, in particular a small number of large, randomized, blinded studies related to the nursing figure who relates to the patient affected by TCM syndrome. In particular, few studies describe nursing differential diagnosis; the long-term outcomes of patients after the nurse’s recognition of the syndrome have not been reported in any of the studies included in this review. However, the role of the nurse in identifying TCM represents a significant theme for future research.

## Conclusions

A proper nursing differential diagnosis and immediate medical treatment can prevent associated complications in TCM, ensuring the best care for patients affected by this interesting “broken heart” syndrome. Our findings confirm this fact and suggest the need for adequate knowledge in nursing education for the identification of TCM from MI, through the specific signs and symptoms that distinguish them. Nonetheless, even today there are few specific educational pathways for nurses, and the role of the Advanced Practice Nurse is still nonexistent in many hospital settings, often accompanied by a total lack of awareness, among the healthcare staff, regarding this syndrome. Moreover, the limited number of on-topic articles found underlines that further research, possibly with large samples, is necessary for a deeper understanding of the nursing role in identifying this condition, and also to better define the types of educational programs that can be developed for the early recognition and identification of TCM.

## Data Availability

Not applicable.
